# Heavy metals in commercial fish and seafood products and risk assessment in adult population in Bosnia and Herzegovina

**DOI:** 10.1038/s41598-020-70205-9

**Published:** 2020-08-06

**Authors:** Jasmina Djedjibegovic, A. Marjanovic, D. Tahirovic, K. Caklovica, A. Turalic, A. Lugusic, E. Omeragic, M. Sober, F. Caklovica

**Affiliations:** 1 Department for Pharmaceutical Analysis, University of Sarajevo-Faculty of Pharmacy, Zmaja od Bosne 8, 71000 Sarajevo, Bosnia and Herzegovina; 2 University of Sarajevo-Veterinary Faculty, Sarajevo, Bosnia and Herzegovina

**Keywords:** Risk factors, Natural hazards

## Abstract

This work investigates the level of exposure to cadmium (Cd), mercury (Hg), and lead (Pb) via fish and seafood products in adult population in Bosnia and Herzegovina (BiH). Metals content was determined in seven commercial species of fish and seafood products widely available and consumed in BiH. Analysis of Cd and Pb was performed by GFAAS (Graphite furnace atomic absorption spectrometry), and analysis of Hg by FIAS AAS (flow injection cold vapour atomic absorption spectrometry) in accredited laboratory for food analysis. The health risk was determined by the estimated weekly intake, hazard index, target hazard quotients, and percent of tolerable weekly intake or percent of benchmark dose lower confidence limit. Concentration above the maximum residue level (MRL) set in the European Union was found in only one sample (Hg in mackerel). Cd content was generally high in squid, approaching the corresponding MRL in two samples. The hazard index was close to 1 in bluefin tuna and mackerel, mostly due to Hg content. These two fish species should be consumed in moderation, especially by pregnant women. While consumption of various fish and seafood on average is not of significant concern, health risk could not be ruled out for high consumers.

## Introduction

Fish is considered a significant part of a healthy, well-balanced diet due to its exceptional nutritional properties (high-quality proteins, vitamins, essential omega-3 fatty acids). Fish and seafood are unique dietary sources of cardioprotective docosahexaenoic (DHA) and eicosapentaenoic (EPA) fatty acids. Thus, many public health authorities recommend regular fish consumption equivalent to at least 1–2 serving per week in order to prevent diet-related chronic diseases^1–3^. Unfortunately, anthropogenic environmental impacts (industry, agriculture, mining) significantly increase the naturally occurring amounts of heavy metals in the environment, including the marine ecosystem. Consequently, marine organisms (fish, shellfish, crustaceans) can accumulate these metals to potentially toxic concentrations. Often, fish and other seafood represent one of the main sources of exposure to metals in the general population. Foods that contain toxic metals above the permitted levels are considered to be harmful to human health and are banned for trade by many national and international regulations. Maximum levels (MRL) of harmful substances in food in Bosnia and Herzegovina (BiH) are defined in the Regulation on maximum levels for certain contaminants in food^4^. The same rules apply to food in the European Union^5–9^. Some of the toxic effects of heavy metals include: impaired renal (Pb, Cd, Hg) and liver (Pb and Cd) function, decreased cognitive function (Pb, Hg), impaired reproductive capacity (Cd, Pb), hypertension (Cd), neurological changes (Hg, Pb), teratogenic effects (Hg), and cancers (Cd)^10–12^. Our previous work showed that the content of heavy metals in certain samples of fish from the Neretva river (BiH) exceeds MRLs for some metals, and content found in many commercially available fish could pose a health risk for high consumers^13^. The MRL value is a single number for a certain pollutant that can only be used to truly determine if the product can be legally traded. However, compliance with these values does not guarantee the safety of the food in case of more frequent consumption. Therefore, risk assessment studies are conducted using different models of food consumption (average intake, lower and upper limits of estimated intake) in different population groups. It is also important to stress out that the compliance with the legal limits (MRL) and food safety, in general, is considered as the responsibility of the food business operator (i.e. producer) by law. With this concept, the official food control has diminished significantly and the number of food samples regularly tested is quite small. On the other hand, independent (academic) research often reveals contaminant levels above the legal limits of randomly selected samples from the market. These data are valuable for both food safety and public health authorities, as can be seen from e.g. the European food safety authority (EFSA) reports on health risk assessment for European consumers, which cite and relies on findings in these research papers.

The aim of this work was to determine toxic metals (Cd, Pb, and Hg) content in different fish and seafood and to assess potential health risk based on previously estimated daily intake in the adult population in BiH^14^. Data on toxic metal concentrations in this group of food from BiH market are scarce and total dietary study is yet to be conducted in BiH. Hence, our results are valuable inputs for food regulatory agency as well as public health authorities.

## Results

The concentration of heavy metals in fish and seafood are presented in Table 1.

**Table 1:** Heavy metal concentrations and maximum residue level—MRL (mg kg^−1^ wet weight) in different fish and seafood species.

Species	Country of origin	n	Cd			Hg			Pb		
			Average ± SD	Range	MRL^a^	Average ± SD	Range	MRL^a^	Average ± SD	Range	MRL^a^
European hake	Spain	3	0.003 ± 0.001	0.002–0.004	0.05	0.023 ± 0.002	0.022–0.025	0.50	0.002 ± 0.001	0.001–0.002	0.30
Atlantic bluefin tuna steak	Portugal	3	0.01 ± 0.0	__	0.10	0.213 ± 0.096	0.114–0.309	1.0	0.003 ± 0.002	0.001–0.004	0.30
Atlantic bluefin tuna (canned)	Thailand	7	0.015 ± 0.003	0.01–0.02	0.10	0.062 ± 0.028	0.037–0.116	1.0	0.006 ± 0.003	0.001–0.008	0.30
Atlantic mackerel	Morocco	5	0.033 ± 0.009	0.021–0.047	0.10	0.192 ± 0.247	0.042–0.624	0.50	0.007 ± 0.005	ND^b^-0.01	0.30
Patagonian squid	Spain	5	0.644 ± 0.252	0.391–0.918	1.0	0.02 ± 0.004	0.014–0.024	0.50	0.003 ± 0.002	0.001–0.006	0.30
Blue mussel	Spain	5	0.062 ± 0.009	0.049–0.073	1.0	0.044 ± 0.011	0.026–0.055	0.50	0.161 ± 0.072	0.092–0.278	1.5
Black tiger shrimp	China	4	0.015 ± 0.002	0.013–0.017	0.5	0.058 ± 0.023	0.029–0.078	0.50	0.014 ± 0.008	< 0.001–0.022	0.5
Indian white prawn	India	5	0.002 ± 0.005	0.015–0.027	0.5	0.037 ± 0.018	0.008–0.056	0.50	0.013 ± 0.008	0.004–0.024	0.5

^a^*MRL* maximum residue level in EU^5–9^.

^b^*ND* not detected.

**Figure 1:**
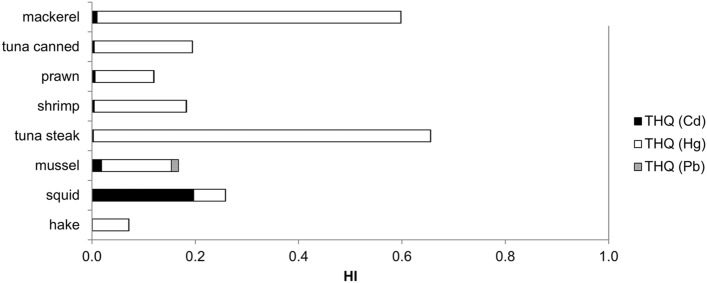
Hazard index (HI) values for one portion of fish or seafood per week.

Hazard index (HI) values for individual species based on minimal recommended consumption of one portion (150 g) of fish or seafood per week are shown in Fig. 1.

**Figure 2:**
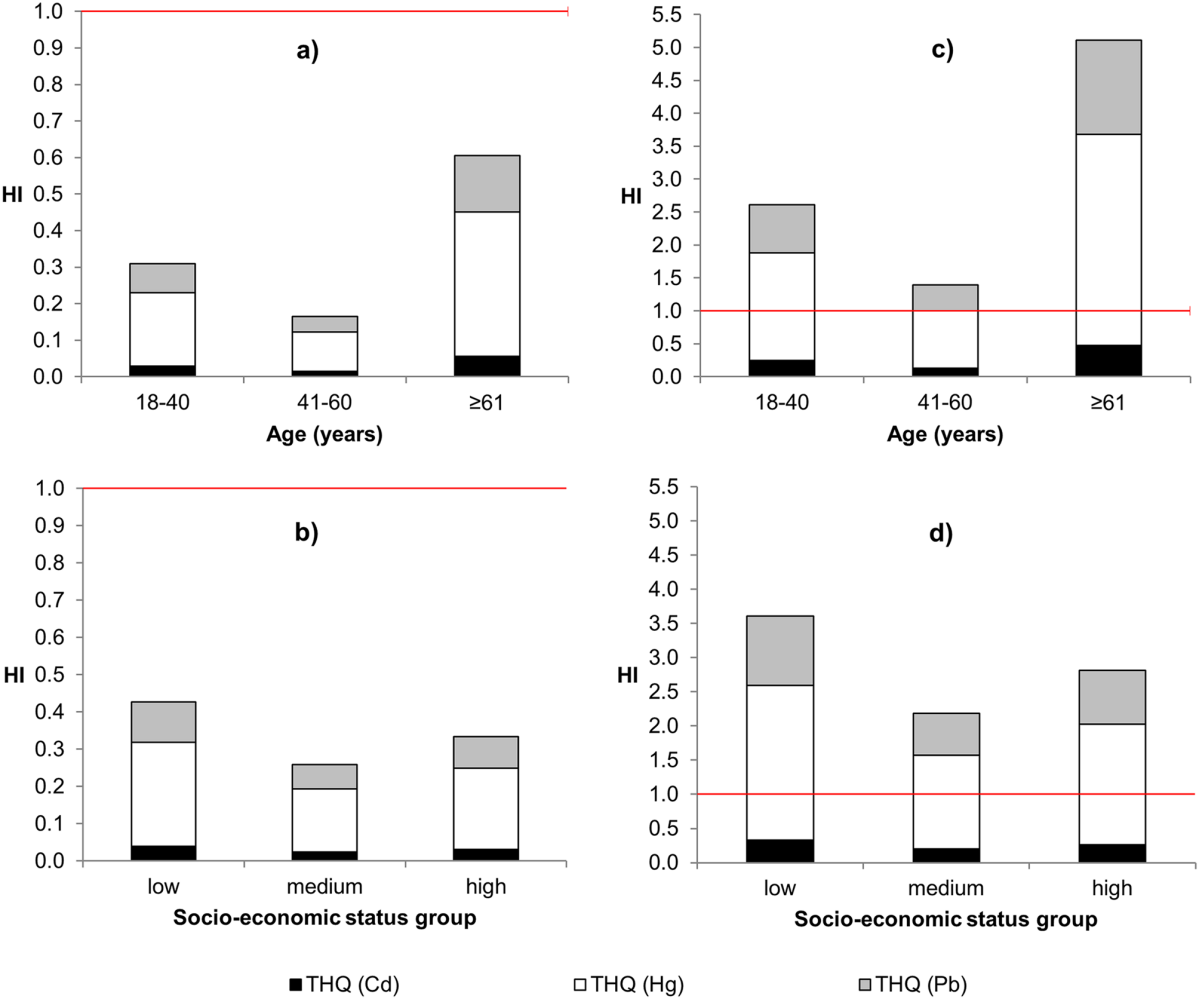
Hazard index (HI) and target hazard quotient (THQ) values by age and socio-economic groups calculate for average fish and seafood intake and geometric mean (**a**,**b**) or maximum (**c**,**d**) metal concentrations.

The THQ (target hazard quotients) and hazard index (HI) values calculated for different age and socio-economic groups are presented in Fig. 2.

Estimated weekly intake of analyzed metals via fish and seafood consumption and %TWI (Tolerable Weekly Intake) for Cd and Hg or %BMDL (Benchmark Dose Lower Confidence Limit) for Pb are presented in Table 2.

**Table 2:** Estimated weekly intake of metals (μg kg^−1^ b.w. and %TWI or %BMDL) based on geometric mean concentration in different age and socio-economic groups.

Weekly intake	Age group (years)			Socio-economic status group		
	18–40	41–60	≥ 61	Low	Medium	High
Cd (μg kg^−1^ b.w.)	0.199	0.106	0.389	0.274	0.166	0.214
Cd (%TWI)	7.95	4.23	15.6	11.0	6.65	8.55
Hg (μg kg^−1^ b.w.)	0.142	0.075	0.277	0.196	0.119	0.152
Hg (%TWI)	10.9	5.80	21.3	15.0	9.12	11.7
Pb (μg kg^−1^ b.w.)	0.055	0.029	0.108	0.076	0.046	0.059
Pb (%BMDL_01_)	3.68	1.96	7.20	5.08	3.08	3.96
Pb (%BMDL_10_)	8.76	4.67	17.1	12.1	7.33	9.43

*TWI* tolerable weekly intake, *BMDL*_*01*_ benchmark dose lower confidence limit (for cardiovascular Pb effects), *BMDL*_*10*_ benchmark dose lower confidence limit (for chronic kidney Pb effects).

## Discussion

Mercury and cadmium were detected in all analyzed samples (100%), while lead was detected in 33 samples (89.2%). Metals content was in order Hg > Cd > Pb in most of the species, except blue mussel (Pb > Cd > Hg) and Indian white prawn (Hg > Pb > Cd). Metals content in the vast majority of samples were well below the MRL (Table 1). Mercury content above the MRL was found in only one sample of Atlantic mackerel (0.624 mg kg^−1^). Cadmium content was close to the corresponding MRL in two samples of Patagonian squid (0.918 and 0.896 mg kg^−1^) and was also quite high in the other three samples of the same species (0.591, 0.425 and 0.391 mg kg^−1^). Cadmium content in Patagonian squid was much higher than in other analyzed species. Similarly, Pb content was much higher in the blue mussel than in other analyzed species. Our results are in good agreement with other published data from the European market^15–17^.

The target hazard quotient (THQ) is a ratio of the potential exposure to a contaminant and the acceptable level of the same contaminant at which no adverse effects are expected (see the “Risk assessment evaluation” for details on the calculation). The hazard index (HI) is computed as the sum of THQs for individual metals and used to assess the total potential health effect due to exposure to a mixture of metals. It is generally accepted that HI > 1.0 indicates that the adverse health effects are possible. Hazard index (HI) calculated on the basis of the mean metal concentration in different species and one portion of fish per week (Fig. 1) was close to 1 for tuna steak (HI = 0.92) and mackerel (HI = 0.84), thus these two species should be consumed in moderation. This should especially be advised to pregnant women (and children) since the main contribution to the overall HI was due to Hg content in both of these species. While most of the food-based dietary guidelines recommend higher fish and seafood intake for pregnant women to provide an adequate dietary intake of DHA and iodine, which have a well-established role in the development of the central nervous system of the fetus, special advice on the type of fish to be limited because of relative high methylmercury content and its neurotoxic effect on fetus are also often provided^18^. Furthermore, the fish to be limited or avoided during pregnancy usually include fresh tuna, while different national guidelines for pregnant women list mackerel as either a safe fish to be eaten freely (e.g. Sweden, Norway, Denmark) or as fish to be limited (e.g. USA, UK, France, Italy)^19^. Our results suggest that both fresh tuna and mackerel consumption should be limited to not more than one portion per week during pregnancy.

Total HI, calculated on the basis of the geometric mean concentrations of metals and mean fish and seafood consumption rate, were below 1 in all consumer groups, with the highest contribution of Hg (Fig. 2a,b). Among different age groups, the highest risk was found for consumers older than 61 years of age (HI = 0.61), followed by group 18–40 years of age (HI = 0.31). In respect to socio-economic status, the highest risk was found for consumers with low status (HI = 0.43), followed by consumers with high status (HI = 0.33). Since at least some of the consumers within groups have higher exposure due to their choice of fish and seafood species, as well as higher than average consumption rate, maximum metals concentrations were used for the worst-case scenario calculation. In this case, the total HI was higher than 1 (Fig. 2), thus health risk could not be neglected in high consumers irrespective of their age or socio-economic status.

Cadmium content (mean concentration) in all analyzed samples would result in a weekly intake of 0.106–0.389 μg kg^−1^ b.w. (Table 2). The contribution to TWI for Cd in the same population groups was 4.23% to 15.6%. Cadmium weekly intake from fish and seafood (fish + molluscs + crustaceans) reported for adult Belgian population in 2010 (0.083 kg^−1^ b.w., 3.33% TWI)^20^ was close to the lowest intake in our study. Considering individual species in our sample, Cd was the critical metal (metal with the highest %TWI) in the Patagonian squid. Based on our results, an adult person (70 kg b.w.) would reach the TWI for Cd byweekly consumption of 272 g of squid, whereas more than tenfold amount of other analyzed species would be needed for the same Cd intake. However, it is important to take into consideration that fish and seafood are usually not one of the major dietary Cd sources. According to EFSA, the main sources of Cd in a diet are the staple foods (wheat, rice, and potatoes), which provide about 40–50% of the ingested metal^20,21^, and habitual tobacco smoking is a significant additional source of this metal^22^. Hence, our data suggest that fish and seafood, especially squid should be considered as relevant dietary Cd sources.

Mercury content (mean concentration) in all analyzed samples would result in a weekly intake of 0.075–0.277 μg kg^−1^ b.w. (Table 2). The contribution to TWI for Hg was 5.80–21.3%. According to European Food Safety Authority (EFSA) report from 2015^23^, estimated mean weekly exposure to Hg from fish in different European countries ranged from < 0.1 (the Netherlands) to 1.6 μg kg^−1^ b.w. (Portugal) (for an adult with b.w. of 60 kg). Our results were within this range and closer to the lower boundary. Mercury was the critical metal in all species except the Patagonian squid and the blue mussel in our sample. An adult person (70 kg b.w.) would reach the TWI for Hg by weekly consumption of 427 g of bluefin tuna steak, 474 g of mackerel, 1.5 kg of canned tuna, 1.6 kg of black tiger shrimp, 2.5 kg of Indian white prawn or 4.0 kg of European hake. Since fish and seafood is virtually exclusive source of methylmercury^24^, these intake rates can be considered as the safe limits for fish and seafood in our sample.

Content (geometric mean concentration) of Pb in our sample corresponds to a weekly intake of 0.029–0.108 μg kg^−1^ b.w. (Table 2). The contribution to BMDL_01_ was 1.96–7.20%, and contribution to BMDL_10_ was 4.67–17.1%. Lead was the critical metal in the blue mussel. This is in accordance with the EFSA report in which bivalve molluscs had the highest incidence of lead contamination in the “fish and seafood” category, with the mean Pb content of about 0.2 mg kg^−1^ (0.161 mg kg^−1^ in our mussel samples)^25^. Based on the mean concentration in our sample, an adult person (70 kg) would reach TWI for Pb by weekly consumption of 274 g of blue mussel. Although EFSA reports that the main sources of Pb exposure for the adult population are food and drinking water, fish and seafood group contributes with only about 1% to the total dietary Pb intake on average, but with considerable variation (between countries and on the individual level) depending on dietary habits^25^. In respect to this, our results suggest that blue mussel could be an important dietary Pb source if consumed regularly.

### Limitations of the study

Although the here presented study is the first of this kind in BiH, it is not without limitations. The authors acknowledge that the sample size was limited and the results should be interpreted as preliminary. The heavy metals intake was estimated based on the concentrations found in a total sample, while the actual intake of various species from this food group was not known. Due to these limitations, as well as natural and expected temporal variations in contaminant levels in food, further studies are needed. The inclusion of a larger sample and survey data on the actual consumption rate of individual species of fish and other seafood is recommended.

## Conclusion

Heavy metals (Cd, Hg, and Pb) were detected in almost all analyzed fish and seafood samples, with the highest concentrations recorded for Hg. The highest HI close to 1 were found for fresh bluefin tuna and canned mackerel, mostly due to Hg. Hence, these two species should be consumed in moderation (not more than one portion per week for pregnant women). For an adult consumer (70 kg b.w.), TWI for Cd and Pb would be reached by weekly consumption of 272 g of squid and 274 g of the blue mussel, respectively. On average, a diet that includes a variety of fish and seafood in observed, as well as recommended consumption rates is unlikely to pose a significant risk due to Cd, Hg and Pb intake in the adult population. The health risk for high consumers could not be ruled out.

Due to the study limitations, these results should be interpreted as preliminary. Further research to additionally validate our results is recommended.

## Matherials and methods

### Sample selection

**Figure 3:**
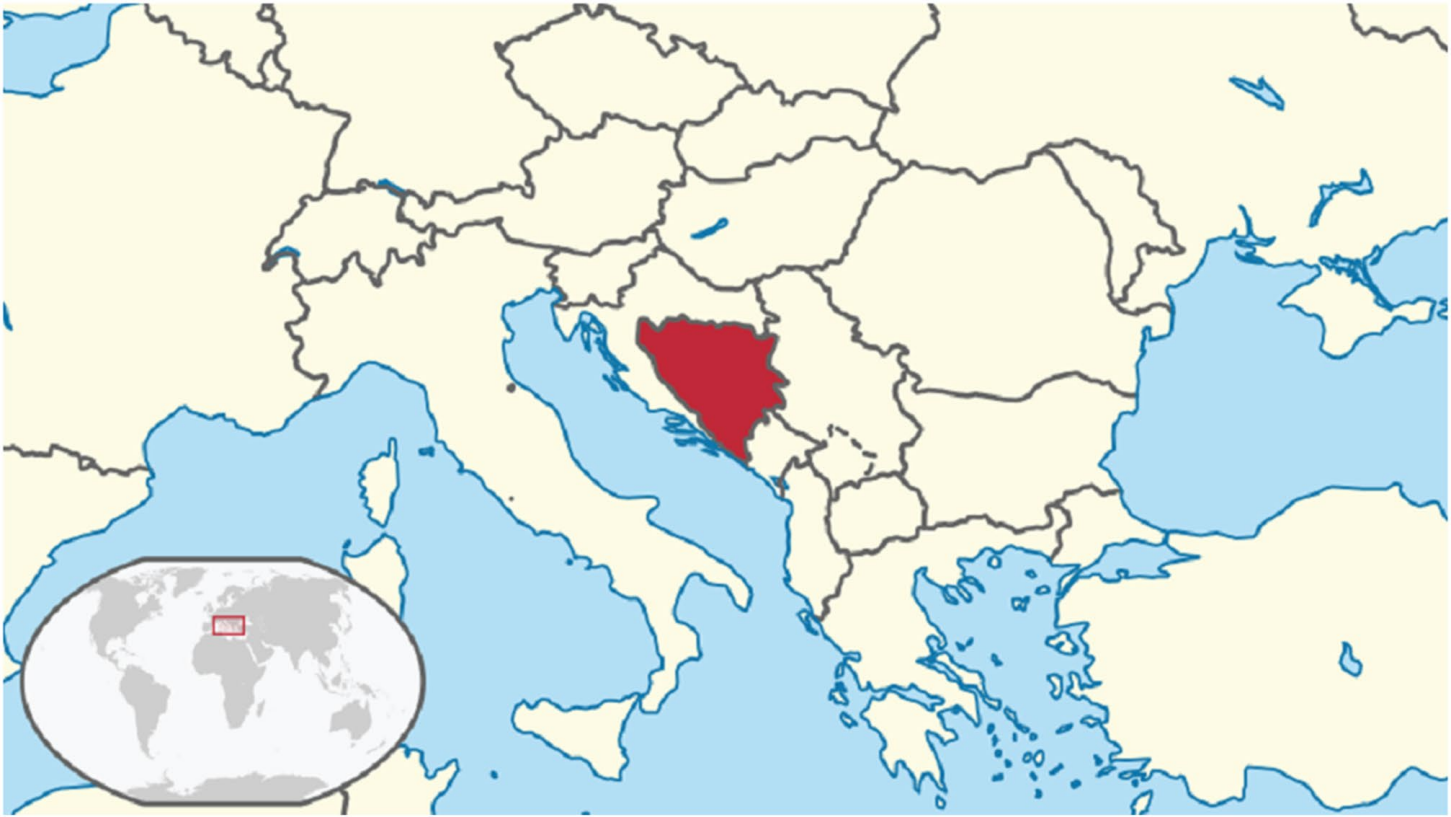
Geographical location of Bosnia and Herzegovina.

Bosnia and Herzegovina (BiH) is a continental country with a narrow exit to the Adriatic Sea (Fig. 3). Despite this fact, most fish and seafood eaten in BiH are bought in grocery stores, usually frozen or canned, probably because it is more easily accessible throughout the year. Thus, we purchased our samples (n = 37) from retail in June 2019. We chose different fish and seafood species widely available and consumed by the local population. The toxic metals content was determined in samples of European hake (*Merluccius merluccius,* Linnaeus 1758)—frozen, Atlantic bluefin tuna (*Thunnus thynnus*, Linnaeus, 1758) – frozen and canned, Atlantic mackerel (*Scomber* *scombrus,* Linnaeus, 1758)—canned, Patagonian squid (*Loligo gahi, *Orbigny, 1835)—frozen, blue mussel (*Mytilus galloprovincialis*, Lamark 1819)—frozen, black tiger shrimp (*Penaeus monodon*, Fabricius 1798)—frozen and Indian white prawn (*Penaeus indicus,* H. Milne Edwards, 1837)—frozen.

### Materials

All chemicals used during the analytical procedure were of ultrapure grade. Nitric acid (68%) and hydrochloric acid (35%) were purchased from Fisher Scientific (UK). Sodium borohydride granules were from Merck Millipore (USA). Matrix modifiers for THGA-AAS (palladium nitrate 2 g/L, magnesium nitrate 10 g/L, and di-ammonium hydrogen phosphate 25 mg/L) were from CARLO ERBA Reagents (Italy). Metals standard solutions (Pb and Cd 1,000 mg/l in HNO_3_ 0.5 mol/l Certipur and Hg 1,000 mg/l in HNO_3_ 2 mol/l Certipur) were from Merck Millipore (USA), and Mussel tissue (elements) ERM certified Reference Material was from Sigma-Aldrich (UK). Samples digestion was performed in the Berghof microwave oven with PTFE vessels.

Ultrapure MilliPore water (Mili-Q Direct 8) was used in all operations. Sample analysis was performed on PinAAcle 900T PerkinElmer AAS with THGA graphite furnace and flow injection for atomic spectroscopy (FIAS) system.

### Sample preparation

The analysis of metals content was performed in an accredited laboratory for food analysis at the Veterinary faculty in Sarajevo, BiH. Samples were prepared according to standards EN 13804:2013^26^ and EN 13805:2014^27^. In brief, an accurately weighted aliquot (0.50 ± 0.1 g) of a homogenized sample (edible part) was transferred in the PTFE vessels for microwave digestion and 6 mL of nitric acid was added. Frozen samples were thawed to room temperature before homogenization. Digestion was performed in the microwave oven by temperature-controlled program: heating to 160 °C for 5 min, holding time 5 min, ramp time 1 min to 190 °C, holding time 15 min, cooling to 100 °C for 10 min. After cooling to room temperature the content of the vessel was transferred to a volumetric flask (20 mL) and diluted with ultrapure water to the mark. This solution was used for the analysis of Pb and Cd, while it was further diluted with 3 mol/L hydrochloric acid (1:10) for determination of Hg content.

### Toxic metals analysis

The content of Pb and Cd was measured by GFAAS (Graphite Furnace Atomic Absorption Spectrometry) with a mixture of matrix modifiers [NH_4_H_2_PO_4_ and Pd(NO_3_)_2_ for Pb; NH_4_H_2_PO_4_ and Mg(NO_3_)_2_ for Cd], according to the EN 14084:2003^28^. The content of Hg was determined by FIAS technique with NaBH_4_ and 3 mol/L HCl according to the in-house validated method (wavelength 253,65 nm; slit 0,7 nm; cell temperature 100 °C; pump speed 120; carrier gas flow 100 ml/min). The content of metals was quantified from the calibration curve.

### Quality control/quality assurance

Quality control was performed by analysis of one aliquot of reference material, as well as one laboratory reagent blank with each batch of samples. All the samples were analyzed in duplicate and metals content was presented as an average. The differences between duplicates were ≤ 6.14%. Analytical method parameters are shown in Table 3. Blank did not contain detectable concentrations of measured metals. The recovery was calculated as the percentage of the true (certified) concentration of a metal in the certified reference material recovered during the analytical procedure. The recovery values were in the range of 80–110% (Table 3), which is acceptable for the levels of the target analytes, indicating the absence of a significant analytical bias.

**Table 3:** Analytical method parameters.

Metal	ERM certified concentration (mg/kg)	Found concentration (mg/kg)	%RSD	LoQ (mg/kg)	Recovery (%)
Cd	0.336	0.30	0.15	0.002	89.3
Hg	0.071	0.072	0.03	0.02	101
Pb	2.18	1.788	0.09	0.01	82.0

*RSD* relative standard deviation, *LoQ* limit of quantification.

Precautionary measures were taken to prevent possible contamination of the samples. All glassware was cleaned by soaking in 1% nitric acid overnight and rinsing with ultrapure water before use.

### Risk assessment evaluation

The risk assessment was estimated based on the Target Hazard Quotient (THQ), hazard index (HI) and contribution to the Tolerable Weekly Intake or Benchmark Dose.

THQ represents the ratio of exposure level to a substance over a specified period to reference dose (RfD) of that particular substance. Thus, THQ ≥ 1 indicates potential health hazards associated with the consumption of certain food. THQ values were calculated by the formula given by U.S. EPA^29^:

1 $$ {\text{THQ}} = \frac{{{\text{EF}} \cdot {\text{ED}} \cdot {\text{FIR}} \cdot {\text{C}}}}{{{\text{RfD}} \cdot {\text{BW}} \cdot {\text{TA}}}} \cdot \mathop {10}\nolimits^{ - 3} $$

where EF is the exposure frequency (365 days year^−1^), ED is the exposure duration equivalent to the average human lifetime (70 years)^30^, FIR is the fish and seafood ingestion rate (g day^−1^), C is the metal concentration in fish tissue (mg kg^−1^), RfD is the oral reference dose for contaminant (mg kg^−1^ day^−1^), BW is the average body weight (70 kg for adults), and TA is the exposure time for non-carcinogens (365 days year^−1^ ED). The oral reference dose for Cd, Hg, and Pb is 1 × 10^–3^, 1 × 10^–4^ and 3.5 × 10^–3^ mg kg^−1^ day^−1^, respectively^31,32^. The RfD value for methylmercury was used since in fish and seafood this metal is almost exclusively present in methylated form (90% of total Hg content)^33^. Since official data on dietary habits of the BiH population is not available, we used the fish and seafood ingestion rates (FIR) reported by Gicevic et al.^14^. They found that mean ingestion rate was 18.4, 9.8, and 36.0 g day^−1^ for age groups 18–40, 41–60 and ≥ 61 years, respectively and 25.4, 15.4, and 19.8 g day^−1^ for socio-economic status groups “low”, “medium” and “high”, respectively. The geometric mean concentrations of analysed metals in all samples (0.108 mg kg^−1^ for Cd, 0.077 mg kg^−1^ for Hg and 0.030 mg kg^−1^ for Pb) and maximum concentrations (0.918 mg kg^−1^ for Cd, 0.624 mg kg^−1^ for Hg and 0.278 mg kg^−1^ for Pb) were combined with different fish and seafood consumption rates reported for different age and socio-economic status groups in order to assess the estimated daily intake of metals.

To evaluate the potential risk of adverse health effects from a mixture of toxic metals the hazard index (HI) was calculated as the sum of THQ for each metal:

2 $$ {\text{HI}} = \mathop {{\text{THQ}}}\nolimits_{{{\text{Cd}}}} + \mathop {{\text{THQ}}}\nolimits_{{{\text{Hg}}}} + \mathop {{\text{THQ}}}\nolimits_{{{\text{Pb}}}} $$

When HI < 1.0, it is unlikely that there will be obvious adverse effects, while HI > 10 indicates high risk and chronic or even acute effect^34^.

Estimated weekly intake of metals via fish and seafood was also compared to corresponding Tolerable Weekly Intake (TWI) for Cd (2.5 μg kg^−1^ b.w.)^21^ and Hg (1.3 μg kg^−1^ b.w.)^18^. Since EFSA and other food safety authorities no longer recommend the use of previously established TWI for Pb, we used two BMDL (Benchmark Dose Lower Confidence Limit) values for Pb: BMDL_10_ (0.63 μg/kg b.w.) and BMDL_01_ (1.5 μg/kg b.w.) for chronic kidney effects and cardiovascular effects^35^. The contribution to the TWI (%TWI) or BMDL (%BMDL) was calculated for mixed seafood consumption using formula:

3 $$ {\text{\% TWI or \% BMDL}} = \frac{{{\text{EWI}}}}{{{\text{TWI}} \cdot {\text{BW}}}} \cdot {100} $$

where EWI is estimated weekly intake of a metal (µg week^−1^), calculated as a product of the geometric mean concentration of each metal (µg g^−1^) and weekly fish and seafood consumption (g).

## Data Availability

The datasets generated and/or analyzed during the current study are available from the corresponding author on reasonable request.
